# CSI 3.0: a web server for identifying secondary and super-secondary structure in proteins using NMR chemical shifts

**DOI:** 10.1093/nar/gkv494

**Published:** 2015-05-15

**Authors:** Noor E. Hafsa, David Arndt, David S. Wishart

**Affiliations:** 1 Department of Computing Science, University of Alberta, Edmonton, AB T6G 2E8, Canada; 2 Department of Biological Sciences, University of Alberta, Edmonton, AB T6G 2E8, Canada

## Abstract

The Chemical Shift Index or CSI 3.0 (http://csi3.wishartlab.com) is a web server designed to accurately identify the location of secondary and super-secondary structures in protein chains using only nuclear magnetic resonance (NMR) backbone chemical shifts and their corresponding protein sequence data. Unlike earlier versions of CSI, which only identified three types of secondary structure (helix, β-strand and coil), CSI 3.0 now identifies total of 11 types of secondary and super-secondary structures, including helices, β-strands, coil regions, five common β-turns (type I, II, I′, II′ and VIII), β hairpins as well as interior and edge β-strands. CSI 3.0 accepts experimental NMR chemical shift data in multiple formats (NMR Star 2.1, NMR Star 3.1 and SHIFTY) and generates colorful CSI plots (bar graphs) and secondary/super-secondary structure assignments. The output can be readily used as constraints for structure determination and refinement or the images may be used for presentations and publications. CSI 3.0 uses a pipeline of several well-tested, previously published programs to identify the secondary and super-secondary structures in protein chains. Comparisons with secondary and super-secondary structure assignments made via standard coordinate analysis programs such as DSSP, STRIDE and VADAR on high-resolution protein structures solved by X-ray and NMR show >90% agreement between those made with CSI 3.0.

## INTRODUCTION

Secondary structures such as α-helices, β-strands and coils are commonly used to describe, understand and visualize protein tertiary structures (1). Because of their importance, the identification and delineation of secondary structure elements has long been an integral part of the protein structure determination process. This is particularly true for nuclear magnetic resonance (NMR)-based protein structure determination where secondary structure is used to help in structure generation and refinement (2,3). In protein NMR, secondary structures are traditionally identified and assigned using NOE-based (Nuclear Overhauser Effect) methods. By manually analyzing the positions and patterns of weak, medium or strong NOEs it is possible to identify helices, β-turns and β-stands with reasonably good accuracy. Even today NOE pattern measurements continue to be the most commonly used method for identifying secondary structures in peptides and proteins (2). However, in addition to NOEs, NMR chemical shifts can also be used to identify secondary structures. The use of chemical shifts to identify protein secondary structures was first demonstrated in the early 1990s with the development of a technique called the Chemical Shift Index or CSI (4). The CSI method applies a three-part or ternary ‘digital filter’ to backbone ^1^H and ^13^C chemical shifts as a way of simplifying the chemical shift information. By comparing the experimentally observed chemical shifts to a set of residue-specific ‘random coil’ chemical shifts and converting significant downfield secondary shifts to ‘1's’, significant upfield secondary shifts to ‘−1's’ and small secondary chemical shifts to ‘0's’, a simple bar graph can be generated. By observing how the 1's or −1's or 0's cluster together in the graph it is possible to accurately identify the type and location of protein secondary structure elements (helices, β-strands, coils) along the length of a protein chain (4,5). The CSI method is particularly popular because it is fast, easy to perform and surprisingly accurate—exhibiting an ∼80% agreement with secondary structures determined from PDB coordinate analysis.

However, the CSI method is not perfect. For instance, it requires nearly complete backbone assignments to obtain good results. Furthermore, it is quite sensitive to the choice of random coil or reference chemical shifts used to calculate the secondary shifts and it tends to be more accurate for helix identification than β-strand identification. Because of these limitations, a number of alternative CSI-like methods have been proposed. These include PSSI (6), PsiCSI (7), PLATON (8), PECAN (9) and 2DCSi (10). Most of these newer methods extend the basic CSI protocol by including more sophisticated chemical shift models or more elaborate statistical calculations. For instance, the developers of PSSI chose to discard CSI's simplistic digital filter and replace it with a sophisticated joint probability model to enhance PSSI's secondary structure identification accuracy. On the other hand, the developers of PsiCSI kept the basic CSI protocol but combined it with a well-known sequence-based secondary structure prediction program called PSIPRED (11) to enhance its performance. In contrast to PSSI and PSIPRED, PLATON uses a database of well-defined reference chemical shift patterns to help identify secondary structures. This pattern database appears to boost its secondary structure identification performance. The program known as PECAN uses a chemical shift ‘energy function’ that combines sequence information with chemical shift information to improve its secondary structure identification accuracy. Finally, 2DCSi uses cluster analysis to extract information for chemical shift scatter diagrams derived from all six backbone chemical shifts to improve its secondary structure identification performance. Most of these methods achieve a three-state secondary structure (Q3) accuracy better than 80%, with some reaching as high as 85%.

Over the past 5 years many chemical shift-based secondary structure assignment methods have begun to exploit machine-learning techniques, torsion angle estimates, sequence similarity assessments, chemical-shift derived flexibility and larger chemical shift-structure databases to improve their performance. These newer methods include TALOS+ (12), TALOS-N (13), DANGLE (14) and CSI 2.0 (15). The TALOS+ and TALOS-N packages use chemical shifts to calculate backbone torsion angles. This information is then used to identify secondary structure locations by exploiting the power of artificial neural networks (ANNs) to match chemical shift patterns against a large database of previously assigned proteins with high-resolution 3D structures. DANGLE employs some of the same concepts found in TALOS+ but instead of ANNs it uses Bayesian-inference techniques to help identify secondary structures. Like the TALOS and DANGLE programs, CSI 2.0 makes use of machine-learning algorithms to integrate multiple pieces of information together but unlike TALOS it also combines more extensive sequence information with additional data regarding chemical-shift-derived flexibility. The performance of these newer ‘shift-to-structure’ programs is now quite impressive with most reporting Q3 accuracies above 85% and with CSI 2.0 achieving a Q3 score of 88–90%. This kind of performance generally exceeds the performance of NOE-only-based methods for secondary structure assignment or identification (15). Furthermore, a Q3 score of 88–90% essentially matches the level of agreement that one achieves by comparing the results of different coordinate-based secondary structure assignment programs such as DSSP (16), STRIDE (17) or VADAR (18) on the same PDB coordinate set (15).

While the performance of the most recent shift-based secondary structure assignment programs is very impressive, they are still missing a significant amount of information that can be easily derived from chemical shifts. This includes such useful information as flexibility, backbone torsion angles and accessible surface area (13,19,20). Furthermore, the traditional 3-state model of secondary structure assignments (helix, β-strand and coil) is often considered rather ‘dated’ and somewhat inadequate with regard to modern expectations of detailed protein topology diagrams or information-rich protein structure descriptions. Three-state secondary structure assignments are also insufficiently precise for many 3D structure generation or 3D structure refinement programs such as XPLOR (21), CYANA (22), CHESHIRE (23), CS-Rosetta (24) and CS23D (25). Ideally if NMR chemical shifts could be used to identify other kinds of secondary or super-secondary structure features such as β-turns, β-hairpins or more complex β-strand topologies then they could be more fully exploited as additional constraints for NMR structure generation and refinement. This same information could also be used to create far more informative protein secondary structure and topology diagrams.

Given the need for this kind of information and given the availability of high performing tools to calculate these features from NMR chemical shift data, we decided to create a new kind of ‘shift-to-structure’ tool. In particular we combined a high-end secondary structure calculation algorithm (CSI 2.0) with a high-performing torsion angle calculator (TALOS-N), an accurate measurement method for backbone flexibility (random coil index (RCI) ) and a robust method for calculating fractional accessible surface areas (fASAs) (Side-chain RCI)—all of which use NMR chemical shifts as input. By linking these four tools together into a single structure determination pipeline and intelligently processing their respective structure assignments we found that it was possible to create a program that accurately identifies 11 types of secondary and super-secondary structures using only backbone NMR chemical shift data. These shift-derived structures include helices, β-strands, coil regions, five common β-turns (type I, II, I′, II′ and VIII), β-hairpins as well as interior and edge β-strands. Since this concept builds from our previous work on the Chemical Shift Index (CSI) and an earlier program called CSI 2.0, we decided to call the new method CSI 3.0. A detailed description of the CSI 3.0 web server along with a discussion of its capabilities and overall performance is given below.

## ALGORITHM AND WORKFLOW

The CSI 3.0 system consists of four well-tested and previously published programs, namely CSI 2.0 (15), TALOS-N (13), RCI (19) and Side-chain RCI (20). CSI 2.0 uses chemical shift and sequence data to accurately identify three types of secondary structures: helices, β-strands and coil regions. Extensive tests have shown that it has a Q3 accuracy (agreement between identified by shifts and those determined by coordinate analysis) of 88–90% depending on the coordinate assignment algorithm that is chosen (15). TALOS-N uses chemical shift and sequence data to calculate backbone torsion angles. It can routinely determine backbone torsion angles for more than 90% of amino acid residues, with a root mean square difference between estimated and X-ray observed (ϕ, ψ) torsion angles of ∼12º (13). The RCI technique uses backbone chemical shifts to calculate the flexibility or order parameters of a protein sequence. The RCI method is frequently used to identify ordered and disordered segments in proteins. The agreement between RCI-calculated order parameters or RMSFs and observed order parameters or RMSFs ranges between 77 and 82% (19). The side-chain RCI or the side-chain RCI is a technique that can be used to calculate residue-specific fASA using side-chain chemical shifts. The original paper reported a correlation coefficient between the shift-calculated fractional ASA and the coordinate measured fASA of ∼0.76 (20). Recent improvements to the algorithm now allow backbone (only) shifts to be used and the correlation between observed and shift-calculated fASAs is now 0.82.

The central concept behind the CSI 3.0 algorithm is to intelligently combine each of the four shift-based calculators into a more comprehensive or more fully integrated structure assignment program that is ‘greater than the sum of its parts’. Specifically by starting with the most accurate method first (secondary structure assignment with CSI 2.0) and then filtering out protein sequence segments that were already assigned a clear secondary structure (helix or β-strand) we found we could selectively apply the less accurate methods (torsion angle, flexibility and fASA calculations) to the remaining regions to identify other secondary or super-secondary structures. For instance, to identify a β-hairpin it is better to start with the precise location of the two sequentially adjacent β-strands and to determine if the ‘coil’ residues between the β-strands have the appropriate torsion angles and sequence characteristics to form a β-hairpin. Similarly, the identification of edge or interior β-strands can only be determined once a β-strand is identified and only then should the fASA, flexibility or other characteristics of the entire β-strand be calculated. Similarly, the identification of β-turns and β-turn types should only be conducted in regions initially identified as ‘coil’ regions (since β-turns are not found in helices or β-strands) and only in regions where the chain is well defined (i.e. an RCI-calculated order parameter >0.7).

**Figure 1.**
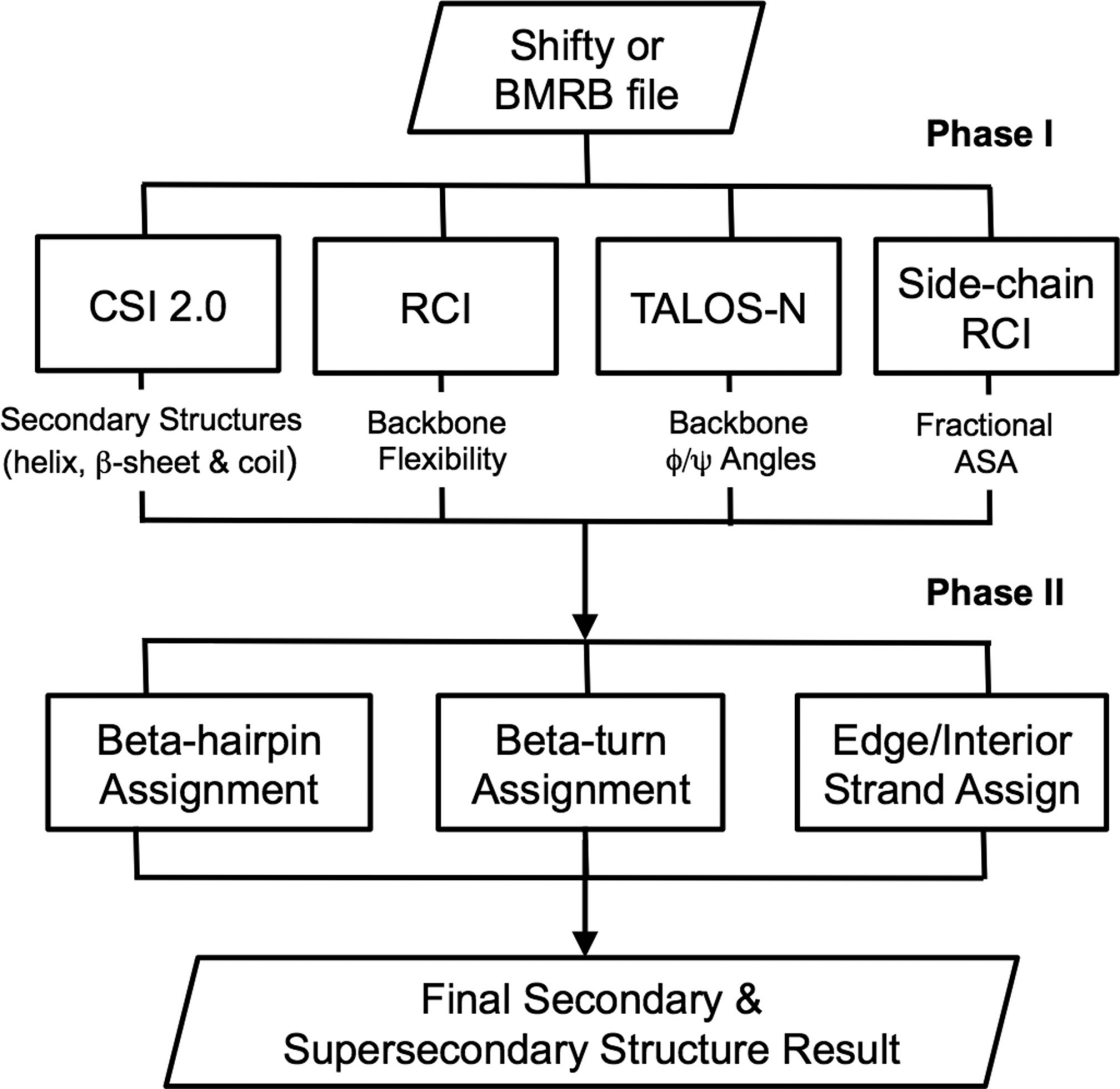
Program flow chart for CSI 3.0.

A flow chart describing the CSI 3.0 algorithm is shown in Figure 1. As can be seen in this diagram the user first provides a file (NMRStar 2.1, NMRStar 3.1 and SHIFTY) containing the protein sequence and the assigned chemical shifts. Complete and properly referenced (26) ^1^H, ^13^C and/or ^15^N chemical shifts are strongly preferred. However ^15^N chemical shift data are not required and the lack of ^15^N shift data typically does not reduce the overall program performance. Once the chemical shift file is provided, CSI 2.0 is called to perform a per-residue three-class secondary structure assignment. Extensive studies have shown that CSI 2.0 is the most accurate method for identifying secondary structures using only chemical shift data (15). Additional details regarding the algorithm and its performance with regard to missing assignments or chemical shift completeness are fully described in the original publication (15). Once the helices, β-strands and coil regions are identified, the RCI program is run. The RCI program calculates backbone flexibility from backbone chemical shifts. Additional details regarding its algorithm, its applications and overall performance are also described in the original publications (19). The purpose of the RCI program is to identify CSI 2.0 annotated coil regions that are too flexible to produce reliable torsion angles (for β-turn identification). Residues that have an RCI-calculated order parameter (S^2^) ≤0.7 are excluded from further analysis. The choice of S^2^ ≤ 0.7 is based on observations from many NMR protein structures that have intrinsically disordered or poorly defined regions. After the RCI filtering step is performed all remaining coil regions have their φ, ψ backbone torsion angles calculated by TALOS-N (13). TALOS-N is widely regarded as the most accurate, shift-based backbone torsion angle calculator. Details of the algorithm and its performance with regard to missing assignments or chemical shift completeness are fully described in the original publication (13). Finally the last program (Side-chain RCI) is used to calculate the fASA for all β-strand residues initially identified by CSI 2.0.

Once the initial per-residue assignment phase (helices, β-strands, coil, order parameters, φ, ψ angles, fASA) has been completed, the algorithm moves to the second phase which involves identifying β-turn types (type I, II, I′, II′ and VIII), β-hairpins and edge/interior β-strands. This ‘contextual assignment’ phase employs the per-residue assignment data from the first phase along with the contextual data from the neighboring residue assignments, local sequence (hydrophobicity) data and additional chemical shift pattern information.

The first part of the contextual assignment phase involves the identification of β-turns. β-turns can be classified into five different types, i.e. type I, II, I′, II′ and VIII, based on the characteristic backbone torsion angles for the central two residue (*i* + 1) and (*i* + 2) locations (27). CSI 3.0's β-turn algorithm scans all regions with two or more consecutive coil assignments having RCI-estimated order parameters >0.7 and compares the TALOS-N estimated torsion angles to those expected for each of the five turn-types. Based on previously published recommendations our algorithm requires that three of the four torsion angles must fall within 30° of their characteristic φ or ψ angles, with one φ/ψ angle allowed to deviate by up to 45° (27). The positional preferences of amino acids in different types of β-turns, which is well known (27), is also used to improve the performance of the algorithm.

The second part of the contextual assignment phase involves identifying β-hairpins. A β-hairpin is formed when a β-turn connects and aligns two anti-parallel β-strands. CSI 3.0's β-hairpin algorithm simply searches for two sequential β-strands that are connected by six or fewer residues containing an appropriate reverse β-turn.

The third part of the contextual assignment phase involves identifying edge (exterior) and interior β-strands. Those β-strands located on the ‘outside’ edges of β-sheets with inter-strand hydrogen bonds only on one side are called edge strands. Those β-strands that have inter-strand hydrogen bonds on both sides are called interior β-strands. Therefore β-sheets with just two β-strands would have two edge strands, β-sheets with three β-strands would have one interior and two edge strands and so on. In general, edge β-strands and interior β-strands are distinguishable by their length (edge strands tend to be shorter), rigidity (interior strands have higher order parameters), repeating patterns of hydrophobic/hydrophilic residues, charged residue distribution, distinct hydrogen bonding patterns and their level of solvent exposure (28)—all of which can be identified via chemical shift data and sequence information. For instance, the differing pattern of hydrogen bonding between edge strands and interior strands generates distinct H^α^ chemical shift patterns. In particular, the H^α^ protons of residues engaged in inter-strand hydrogen bonds tend to be deshielded, leading to downfield secondary chemical shifts. On the other hand, the H^α^ protons of residues that are only hydrogen bonded to water (i.e. edge) tend to be shielded, leading to slight upfield or far weaker downfield secondary chemical shifts. Therefore an alternating pattern of upfield/downfield secondary chemical shifts is often seen in edge strands. This pattern was also noted by others as early as 1994 (29). An interior β-strand, on the other hand, will not exhibit this pattern. CSI 3.0's edge-strand detection algorithm uses a simple pattern matching routine to identify the characteristic alternating H^α^ chemical shift patterns of candidate edge strands. Those that exceed the threshold are given an edge-strand score of 1 (which is added to other evidence to determine the presence of an edge strand).

In addition, to these distinct chemical shift patterns, residues in edge β-strands tend to have greater average accessible surface area (fASA) than interior β-strands, which are usually buried in the protein core. Because the fASA of a residue can be reasonably well determined by its chemical shifts, we used the shift-derived fASA to calculate the average exposure of each β-strand. Those strands with an average strand fASA > 0.3 are given an edge-strand score of 1. Edge strands also exhibit an alternating pattern of exposed and buried residues. For CSI 3.0 we again use the shift-derived fASA information to assign all β-strand residues into one of the two categories, either exposed or buried (a fASA > 0.25 identifies exposed residues). After this categorization is done, CSI 3.0's edge-strand detection algorithm calculates the fraction of exposed residues along the length of each strand. Those β-strands that have a majority (>0.50) of exposed residues are given an edge-strand score of 1.

Because interior strands tend to be more rigid, they often have comparatively higher S^2^ order parameters than edge strands. Therefore we calculated the fraction of ‘rigid’ residues in each strand (an RCI-calculated parameter (S^2^) >0.90). Those β-strands with a total fraction of rigid residues <0.40 were given an edge-strand score of 1. Likewise edge β-strands are often characterized by a pattern of alternating hydrophilic and hydrophobic residues. CSI 3.0's edge-strand detection algorithm uses a simple formula (28) to detect this periodicity and those that exceed the matching threshold are given an edge-strand score of 1. Interestingly, edge strands are often characterized by a higher proportion and a more central positioning of charged residues along the strand (28). For example, charged residues are often found at the middle of an edge strand, whereas they are almost never found in the middle of an interior strand. The proportion and position of charged residues is calculated as described earlier (28) and those exceeding the threshold score are given an edge-strand score of 1. As observed by many others, the length of edge strands tends to be much shorter than interior strands, consequently all short (<5 residues) β-strands are given an edge strand score of 1. All of the edge-strand scores for each β-strand are then added together and those β-strands that exceed a final value of 4 are assigned by CSI 3.0 as an edge strand. All remaining strands are identified as interior strands.

## RESULTS AND VALIDATION

To demonstrate the utility of CSI 3.0 we evaluated its performance for both secondary and super-secondary structure identification using a set of 13 proteins with known 3D structures. The proteins were chosen to span a broad range of sizes (50–200 residues), secondary structure content, turn types, super-secondary structure features and 3D folds. These proteins also had an average level of backbone chemical shift completeness of 95% (which is relatively high). For each of the corresponding 3D structures, the identification of the consensus secondary structures (type and location) was performed by carefully combining the DSSP, STRIDE, VADAR and author assignments together (16–18). For this particular evaluation β-strands (more formally known as β-bridges) with two or fewer residues were classified as coil regions. The identification of β-hairpins as well as the identification of edge and interior β-strands was done through visual inspection of the 3D structures using PyMOL. The complete set of proteins along with their consensus 3D structural assignments (as well as with their CSI 3.0 identified structural elements) is available on the CSI 3.0 website. For the entire set of 13 proteins there were 444 residues in helices, 349 residues in β-strands and 635 residues in coil regions. Within the coil regions there were 160 residues in type I turns, 12 residues in type I′ turns, 36 residues in type II turns, eight residues in type II′ turns and four residues in type VIII turns. Additionally there were a total of 14 β-hairpins, 31 edge β-strands and 28 interior β-strands. Note that only regions that are well defined (as identified by the RCI-derived order parameter score >0.7) and which had non-overlapping β-turns were used in the evaluation of the β-turn performance. The evaluation metric for the secondary structures (i.e. helices, β-strands, coils) was the standard Q3-score evaluated over all residues. The evaluation metric for β-turns (type I, I′, II, II′,VIII β-turns and non-turns) was a simple Q6 score evaluated over all residues. The evaluation metric for the β-hairpins (hairpins and non-hairpins) was a Q2 score while the evaluation metric for the edge, interior and non-edge/non-interior strands was a Q3 score. The ‘Q*n*’ score is essentially a percent correct score similar to a multiple-choice exam where *n* is the number of possible answers for each question. The results are shown in Table 1.

**Table 1.** Performance evaluation of CSI 3.0 on 13 selected proteins

Protein ID	Number of residues	Q3 score (H,B,C)	Q6 score (I,I′,II,II′,VIII turns, non-turns)	Q3 score (edge/interior β-strand, non-strand)	Q2 score (β-hairpins, non-hairpins)
Ubiquitin (Human) PDB: 1UBQ; BMRB: 5387	76	99	100	98	99
GB1 domain (*Streptococcus*) PDB: 1GB1; BMRB: 7280	58	96	100	91	96
Parvalbumin (Human) PDB: 1RK9; BMRB: 6049	110	97	100	100	95
Dinitrogenase (Thermotoga Mortima) PDB: 1O13; BMRB: 6198	124	97	100	85	97
Cyclic nucleotide protein (M. loti) PDB:1VP6; BMRB:15249	142	98	100	90	94
Glutaredoxin (Poxvirus) PDB: 2HZE; BMRB: 4113	108	100	100	96	100
SH3 domain Myo3 (Yeast) PDB: 1RUW; BMRB:6197	70	100	97	80	100
Acyltransferase (*Arabidopsis thaliana*) PDB: 1XMT; BMRB: 6338	103	96	100	93	98
Cytosine Deaminase (Yeast) PDB: 1YSB; BMRB: 6223	158	96	100	95	NA
Sortase A (*Staphylococcus*) PDB: 1T2W; BMRB:4879	148	91	97	85	95
Peptidyl-tRNA hydrolase (*Mycobacterium tuberculosis*) PDB: 2Z2I; BMRB: 7055	191	96	97	97	100
Photoactive Yellow Protein (*Hoeflea halophila*) PDB: 1ODV; BMRB: 6321	100	100	96	80	100
Calmodulin (Bovine) PDB: 1A29; BMRB: 547	148	96	100	98	NA

This table shows that, as expected, the agreement between the per-residue secondary structure assignments derived by chemical shifts matches very well to those determined by analyzing the coordinate data. The average Q3 score for the three main secondary structure types was 97%, which actually exceeds the performance of other state-of-the-art chemical shift-based methods. The average agreement between the observed structure and the CSI 3.0 identified structure was 98% for helices, 96% for β-strands and 96% for coil regions (prior to β-turn ID). Likewise the average Q6 score for β-turns/non-turns was 99%, with a range spanning between 98% (type I) and 100% (type II, I′, II′, VIII). In terms of the super-secondary structure identification (edge, interior and β-hairpins), the average Q2 score for CSI 3.0 for hairpins/non-hairpins was 98%. For edge/interior β-strands/non-strands, the average Q3 score was 91%. CSI 3.0 achieved an edge β-strand accuracy of 73%, an interior β-strand accuracy of 88% and a non-strand accuracy of 97%. Closer inspection of the results showed that the disagreements between 3D structure-generated assignments and those derived by CSI 3.0 were often ambiguous or ‘close calls’. This was particularly true with regard to the identification of edge strands. In many cases edge and interior β-strands have a ‘dual’ nature with some regions of any given β-strand being exposed and others being hydrogen bonded. In certain cases, it appears that CSI 3.0 struggled with identifying these hybrid β-strands. However, it is important to remember that this level of topological information is rarely obtained from preliminary NOE data or NOE pattern matching methods and is often not revealed until the final 3D structure is generated and thoroughly refined. Overall, we believe CSI 3.0's level of performance greatly exceeds what is achievable from NOE pattern matching methods and it is certainly sufficient to provide a useful topologically rich picture of protein structures (for illustrative or publication purposes) and to provide useful constraint data that could be used to generate and refine 3D protein structures using additional NOE or chemical shift (only) data from any number of packages.

## WEB SERVER IMPLEMENTATION

**Figure 2.**
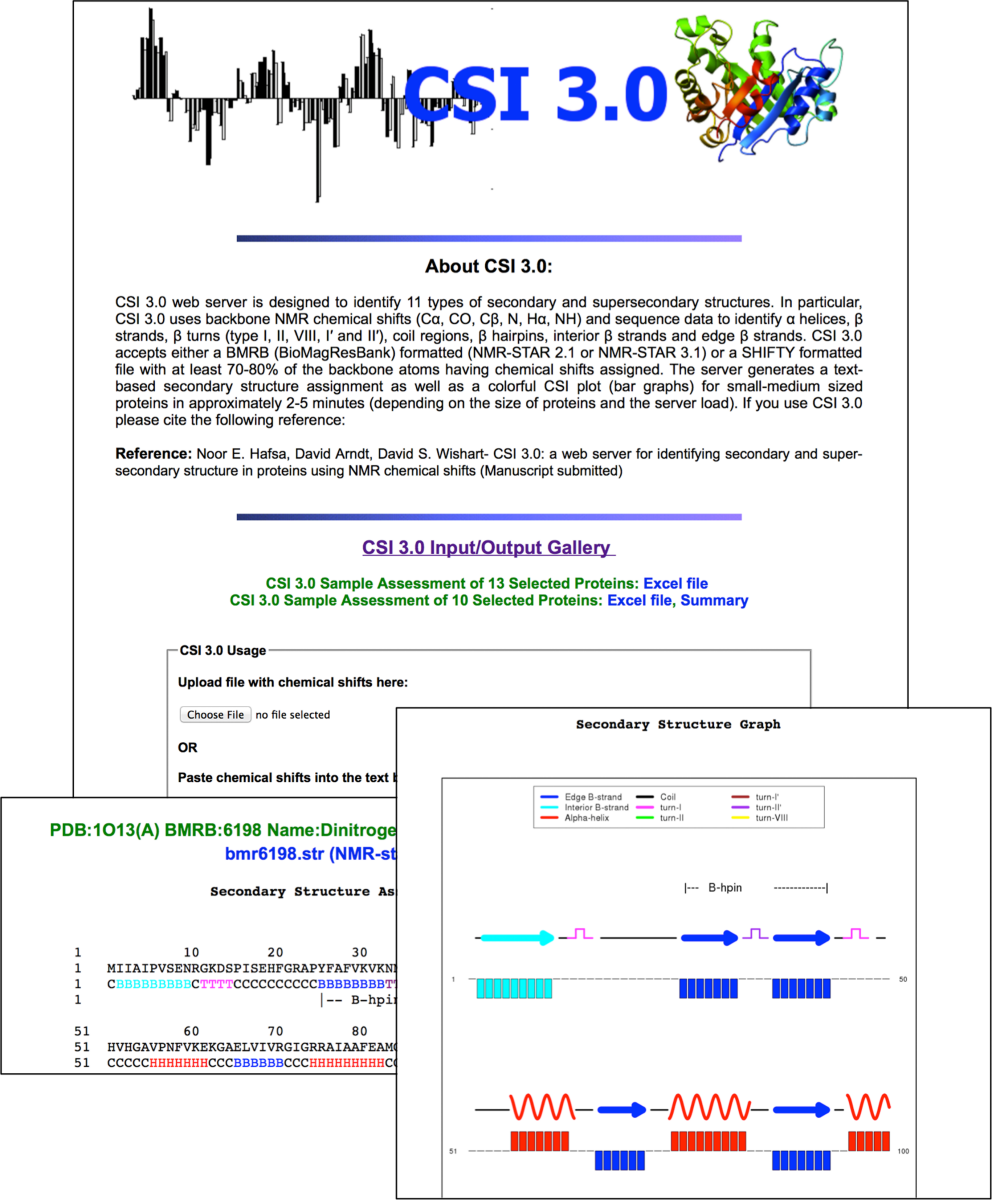
A montage of the CSI 3.0 web server and typical output screenshots.

In developing the CSI 3.0 server we endeavored to create a simple graphical interface that allows users to submit experimental NMR chemical shift data (from a single contiguous polypeptide) by either uploading the files or pasting them into a text box. Multiple chemical shift assignment formats (NMRStar 2.1, NMRStar 3.1 and SHIFTY) are accepted and examples of these formats are provided on the website. After submitting the shift file, the server generates colorful CSI plots or bar graphs (generated by the R package, version 3.0.2) with annotated helices, strands and color-coded indications of β-turns, β-hairpins or edge and interior β-strands. The images are available in a BMP format. A text file with the secondary and super-secondary structure assignments is also generated. The CSI 3.0 web server has been implemented as a Python CGI script (v. 1.1). The component programs were written in Python (CSI 2.0, Side-chain RCI, RCI) or in C++ (TALOS-N). The web application is platform-independent and has been tested successfully under Linux, Windows and Mac operating systems. CSI 3.0 has also been tested and found to be compatible with most modern web browsers including: Google Chrome (v. 31 and above), Internet Explorer (v. 9 and above), Safari (v. 7 and above) and Firefox (v. 23 and above). In general, the CSI 3.0 web server takes 2–5 min to complete its calculations, depending on server load and the length of the protein query. The server is freely available at http://csi3.wishartlab.com. A montage view of the CSI 3.0 web server along with screenshot examples of its output is shown in Figure 2.

## CONCLUSION

CSI 3.0 is an accurate, automated, easy-to-use web service for calculating structural information from chemical shift data. In particular, CSI 3.0 accurately determines eight types of local secondary structures (helices, β-strands, coils and five types of β-turns) as well as three types of super-secondary structures or topological features (β-hairpins, edge strands and interior strands). This represents nearly a 4-fold increase in the number of secondary structure types identified by any other shift-analysis tool that we are aware of—including its predecessor, CSI 2.0. We believe that the additional secondary structure data along with the useful topological information and colorful graphical output generated by CSI 3.0 will not only improve the quality of preliminary protein structure descriptions (often obtained shortly after chemical shift assignments are completed) but also facilitate protein structure determination by NMR. In particular, with the recent trends toward protein structure determination and refinement using chemical shifts (only), chemical shift threading or minimal numbers of NOEs, this added information could prove to be particularly useful to a growing number of NMR spectroscopists.

## References

[1] Richardson J.S. (1981). The anatomy and taxonomy of protein structure. Adv. Protein Chem..

[2] Wuthrich K. (1986). NMR of Proteins and Nucleic Acids.

[3] Wuthrich K. (1990). Protein structure determination in solution by NMR spectroscopy. J. Biol. Chem..

[4] Wishart D.S., Sykes B.D., Richards F.M. (1992). The chemical shift index: a fast and simple method for the assignment of protein secondary structure through NMR spectroscopy. Biochemistry.

[5] Wishart D.S., Sykes B.D. (1994). The 13C chemical shift index: a simple method for the identification of protein secondary structure using 13C chemical shift data. J. Biomol. NMR.

[6] Wang Y., Jardetzky O. (2002). Probability-based protein secondary structure identification using combined NMR chemical-shift data. Protein Sci..

[7] Hung L.H., Samudrala R. (2003). Accurate and automated classification of protein secondary structure with PsiCSI. Protein Sci..

[8] Labudde D., Leitner D., Krüger M., Oschkinat H. (2003). Prediction algorithm for amino acid types with their secondary structure in proteins (PLATON) using chemical shifts. J. Biomol. NMR.

[9] Eghbalnia H.R., Wang L., Bahrami A., Assadi A., Markley J.L. (2005). Protein energetic conformational analysis from NMR chemical shifts (PECAN) and its use in determining secondary structural elements. J. Biomol. NMR.

[10] Wang C.C., Chen J.H., Lai W.C., Chuang W.J. (2007). 2DCSi: identification of protein secondary structure and redox state using 2D cluster analysis of NMR chemical shifts. J. Biomol. NMR.

[11] Jones D.T. (1999). Protein secondary structure prediction based on position-specific scoring matrices. J. Mol. Biol..

[12] Shen Y., Delaglio F., Cornilescu G., Bax A. (2009). TALOS+: a hybrid method for predicting protein backbone torsion angles from NMR chemical shifts. J. Biomol. NMR.

[13] Shen Y., Bax A. (2013). Protein backbone and side chain torsion angles predicted from NMR chemical shifts using artificial neural networks. J. Biomol. NMR.

[14] Cheung M.S., Maguire M.L., Stevens T.J., Broadhurst R.W. (2010). DANGLE: a Bayesian inferential method for predicting protein backbone dihedral angles and secondary structure. J. Magn. Reson..

[15] Hafsa N.E., Wishart D.S. (2014). CSI 2.0: a significantly improved version of the Chemical Shift Index. J. Biomol. NMR.

[16] Kabsch W., Sander C. (1983). Dictionary of protein secondary structure: pattern recognition of hydrogen-bonded and geometrical features. Biopolymers.

[17] Frishman D., Argos P. (1995). Knowledge-based protein secondary structure assignment. Proteins.

[18] Willard L., Ranjan A., Zhang H., Monzavi H., Boyko R.F., Sykes B.D., Wishart D.S. (2003). VADAR: a web server for quantitative evaluation of protein structure quality. Nucleic Acids Res..

[19] Berjanskii M.V., Wishart D.S. (2005). A simple method to predict protein flexibility using secondary chemical shifts. J. Am. Chem. Soc..

[20] Berjanskii M.V., Wishart D.S. (2013). A simple method to measure protein side-chain mobility using NMR chemical shifts. J. Am. Chem. Soc..

[21] Schwieters C.D., Kuszewski J.J., Tjandra N., Clore G.M. (2003). The Xplor-NIH NMR molecular structure determination package. J. Magn. Reson..

[22] Guntert P. (2004). Automated NMR structure calculation with CYANA. Methods Mol. Biol..

[23] Cavalli A., Salvatella X., Dobson C.M., Vendruscolo M. (2007). Protein structure determination from NMR chemical shifts. Proc. Natl. Acad. Sci. U.S.A..

[24] Shen Y., Lange O., Delaglio F., Rossi P., Aramini J.M., Liu G., Eletsky A., Wu Y., Singarapu K.K., Lemak A. (2008). Consistent blind protein structure generation from NMR chemical shift data. Proc. Natl. Acad. Sci. U.S.A..

[25] Wishart D.S., Arndt D., Berjanskii M., Tang P., Zhou J., Lin G. (2008). CS23D: a web server for rapid protein structure generation using NMR chemical shifts and sequence data. Nucleic Acids Res..

[26] Wishart D.S., Case D.A. (2001). Use of chemical shifts in macromolecular structure determination. Methods Enzymol.

[27] Hutchinson E.G., Thornton J.M. (1994). A revised set of potentials for β-turn formation in proteins. Protein Sci..

[28] Siepen J.A., Radford S.E., Westhead D.R. (2003). β-edge strands in protein structure prediction and aggregation. Protein Sci..

[29] Ösapay K., Case D.A. (1994). Analysis of proton chemical shifts in regular secondary structure of proteins. J. Biomol. NMR.

